# Novel Sequential Screening and Enhanced Production of Fibrinolytic Enzyme by* Bacillus* sp. IND12 Using Response Surface Methodology in Solid-State Fermentation

**DOI:** 10.1155/2017/3909657

**Published:** 2017-01-17

**Authors:** Ponnuswamy Vijayaraghavan, P. Rajendran, Samuel Gnana Prakash Vincent, Arumugaperumal Arun, Naif Abdullah Al-Dhabi, Mariadhas Valan Arasu, Oh Young Kwon, Young Ock Kim

**Affiliations:** ^1^Centre for Marine Science and Technology, Manonmaniam Sundaranar University, Rajakkamangalam, Kanyakumari District, Tamil Nadu 629 502, India; ^2^Kanyakumari Field Centre, Central Marine Fisheries Research Institute, Kanyakumari, Tamil Nadu 629702, India; ^3^Department of Biotechnology, Kalasalingam University, Srivilliputtur, Virudhunagar, Tamil Nadu 626126, India; ^4^Department of Botany and Microbiology, Addiriyah Chair for Environmental Studies, College of Science, King Saud University, P. O. Box 2455, Riyadh 11451, Saudi Arabia; ^5^Department of Medical Education and Medical Humanities, School of Medicine, Kyung Hee University, Seoul, Republic of Korea; ^6^Department of Medicinal Crop Research, RDA, Eumseong, Chungbuk 369-873, Republic of Korea

## Abstract

Fibrinolytic enzymes have wide applications in clinical and waste treatment. Bacterial isolates were screened for fibrinolytic enzyme producing ability by skimmed milk agar plate using bromocresol green dye, fibrin plate method, zymography analysis, and goat blood clot lysis. After these sequential screenings,* Bacillus* sp. IND12 was selected for fibrinolytic enzyme production.* Bacillus* sp. IND12 effectively used cow dung for its growth and enzyme production (687 ± 6.5 U/g substrate). Further, the optimum bioprocess parameters were found out for maximum fibrinolytic enzyme production using cow dung as a low cost substrate under solid-state fermentation. Two-level full-factorial experiments revealed that moisture, pH, sucrose, peptone, and MgSO_4_ were the vital parameters with statistical significance (*p* < 0.001). Three factors (moisture, sucrose, and MgSO_4_) were further studied through experiments of central composite rotational design and response surface methodology. Enzyme production of optimized medium showed 4143 ± 12.31 U/g material, which was more than fourfold the initial enzyme production (978 ± 36.4 U/g). The analysis of variance showed that the developed response surface model was highly significant (*p* < 0.001). The fibrinolytic enzyme digested goat blood clot (100%), chicken skin (83 ± 3.6%), egg white (100%), and bovine serum albumin (29 ± 4.9%).

## 1. Introduction

Fibrinolytic enzymes are used as thrombolytic agents to treat cardiovascular diseases and stroke. The plasminogen activator molecules, namely, urokinase, streptokinase, and tissue plasminogen activator, are commonly used in thrombolytic therapy but are expensive and also have unintended physiological effects like bleeding complication, low fibrin specificity, and short half-lives [1]. Hence, the search for the effective and safe thrombolytic agent from different biosources keeps growing worldwide. Study reports are available on fibrinolytic enzymes from various organisms, including* Bacillus polymyxa* [2],* Escherichia coli* [3],* Pseudomonas* [4],* Streptomyces* [5],* Paenibacillus* sp. IND8 [6], and* Bacillus cereus* IND1 [7]. Fibrinolytic enzymes have the ability to inhibit blood coagulation and are able to degrade the fibrin [8]. These enzymes have been reported to be produced in both solid-state fermentation (SSF) and submerged fermentation processes.

SSF has emerged as a useful method for production of enzymes and many significant pharmaceutical products. In a SSF process, usage of convenient substrate is one of the important factors and the yield is considerably higher than submerged fermentation [9]. Agroindustrial residues like wheat bran [9], pigeon pea [10], potato peel [11], grass powder [12], and chicken feather [13] were used for the production of enzymes. Apart from these agroindustrial wastes, researchers attempted to use various effluents including dairy and tannery industrial effluents for the production of proteolytic enzymes. These industrial wastes are rich in nitrogen and carbon sources. Several studies have proved the potential use of these industrial wastes to minimize pollution and to increase the product yield [14, 15]. The marine food waste, shrimp peptone, was also shown to be a potential substrate for SSF involving the production of proteases, recently [16]. The agricultural wastes such as peanut shell, corncob, pomace of corn, and peels of cassava were used for the production of tetracycline [17]. The antibiotic, Cephamycin C, was produced in SSF using wheat straw, deoiled cake of cotton seed, sunflower cake, and corn steep liquor [18] and wheat bran and sweet lemon peel was used as the substrate for the production of nigerloxin in SSF [19]. Apple pomace and chickpeas were used as the substrate for fibrinolytic enzyme production in SSF by* Bacillus cereus* GD55 [20] and* Bacillus amyloliquefaciens* [21].

Generally, enzyme production in a bioprocess is significantly influenced by medium components such as nitrogen and carbon sources and physical factors such as inoculum, pH, temperature, and incubation time [22]. The cost of medium components is one of the critical factors in an industrial perspective, because approximately one-third of the production cost of enzyme was attributed to growth medium cost [23]. Hence, the utilization of locally available inexpensive substrates could bring down the production cost [24]. Medium optimization is also an important factor in enzyme bioprocess. Optimized media enhance the enzyme yield and reduce the production cost. Optimization of process parameters through a one-factor-at-a-time approach is useful; however, it fails to provide the interactions between the variables or factors. The effective method is statistical optimization wherein the medium components having the most significant effect on bioprocess can be identified and optimized [25].

Central composite rotational design (CCRD) and response surface methodology (RSM) elucidate the best-yielding concentration of each factor in the fermentation process. The number of variables that can be studied with lower number of experiments is more in RSM. This saves cost and time and makes RSM more effective than classical methods [26]. RSM has been applied to various cases of enzyme production [27–29]. In recent years, proteolytic enzymes have attracted much more attention for their hydrolytic activity toward recalcitrant animal proteins such as keratin [30] and amyloid prion proteins [31] and collagen [32] and blood clot [33] and management of industrial and medical wastes. Screening of the significant factors was elucidated by two-level full-factorial design and optimization of the significant variables using RSM [25]. Bacterial fibrinolytic enzymes, especially* Bacillus* sp., can be effectively utilized to cure or prevent cardiovascular diseases [34]. In the present study, sequential optimization strategy was followed to enhance fibrinolytic enzyme production from* Bacillus* sp. IND12 utilizing cow dung as the solid substrate.

## 2. Materials and Methods 

### 2.1. Screening

**Step 1 (screening of protease-producing organisms).**

Soil, fish, and rice were used as the sample sources for the production of fibrinolytic enzyme. Soil sample was collected from an agricultural field. Rice was boiled for 45 min and allowed for aerobic fermentation for a period of 48 h at room temperature (30 ± 2°C) and used as the source of bacteria. The marine fish,* Sardinella longiceps*, was collected from Kanyakumari Coast, Tamil Nadu, India, for the isolation of marine bacterial isolates. One gram of sample was transferred to an Erlenmeyer flask (100 mL) with 50 mL of double-distilled water. It was shaken for 10 min and this was resuspended in distilled water and aliquots were spread on skimmed milk agar plates (skimmed milk, 10; beef extract, 1.5; peptic digest of animal tissue, 5.0; yeast extract, 1.5; sodium chloride, 5.0; agar, 1.5; bromocresol green, 0.015 [g/L], pH, 7.0) [35]. For marine isolates, 3% (v/w) sodium chloride was added with the culture medium. The potent proteolytic enzyme-producing bacterial isolate was calculated on the basis of *P*_*z*_ value as described by Price et al. [36]. The bacterial isolate with low *P*_*z*_ value was considered for optimization of enzyme production.

*P*_*z*_ value was calculated by using the following equation:

(1) $$\begin{array}{c} P_{z}=\frac{\text{Colon diameter}}{\text{Colon diameter}+\text{Zone of precipitation}}. \end{array}$$

The isolated protease-producing bacteria were cultured individually in nutrient broth medium.

This was performed by inoculating 1% (v/v) precultured protease positive isolates and incubated at 37°C for 48 h. The cell-free supernatant was used as the source of fibrinolytic enzyme.

**Step 2 (screening of fibrinolytic enzyme-producing bacterial isolates (secondary screening)).**

Fibrinolytic activity of the crude enzyme was tested in a fibrin plate composed of 0.1 M sodium phosphate buffer (pH 7.4), 1% (w/v) agarose, 1.2% (v/v) fibrinogen, and thrombin (100 NIH units/mL). The fibrin plate was allowed to stand for 1 h at room temperature to form a fibrin clot layer. About 20 *μ*L of crude enzyme was dropped into holes and incubated at 37°C for 5 h. The fibrinolytic enzyme exhibited a clear zone of degradation of fibrin around the well, thus indicating its fibrinolytic activity [37]. Ten organisms were screened, and potent organism was retained for this study. The potent isolate was subjected to biochemical characterization and 16S rDNA sequencing.

**Step 3 (fibrin zymography).**

Fibrin zymography was performed to determine fibrinolytic activity of protein having different molecular weight. Bovine fibrinogen (0.12%) and bovine thrombin (10 NIH U/mL) were copolymerized with 12% (w/v) sodium dodecyl sulfate- (SDS-) polyacrylamide gel. After the electrophoresis run, the gel was incubated in 50 mM Tris buffer (pH 7.4) containing 2.5% (v/v) Triton X-100 and incubated in zymogram reaction buffer (Tris buffer, pH 7.4, and 50 mM) for 5 h at 37°C. At the last step, the gel was subjected to staining with Coomassie Brilliant Blue R-250 for a period of 1 h, after which it was destained and bands corresponding to fibrinolytic activities were observed as the unstained regions [38].

**Step 4 (in vitro blood clot lytic activity of fibrinolytic enzymes).**

The clot lytic activity was studied by incubating the crude extract with goat blood in vitro. The goat blood clot was severed into small pieces (400 ± 50 mg), and 200 U streptokinase (positive control), sodium phosphate buffer (negative control), and 200 U fibrinolytic enzyme were added. Then the mix was incubated at 30 ± 2°C for 24 h, and the results were observed. Based on primary and secondary screening, fibrin zymography, and in vitro blood clot lytic activity of the crude fibrinolytic enzyme, the potent organism was selected for further studies.

### 2.2. Identification of Potent Strain

The screened bacterial strain IND12 with highest fibrinolytic enzyme activity was identified based on biochemical characters [39] and 16S rRNA gene sequencing [40]. The genomic DNA of the strain IND12 was extracted by using a QIAGEN kit (Germany). The 16S rRNA gene of the strain was amplified using the upstream primer (P1: 5′-AGAGTTTGATCMTGGCTAG-3′) and the downstream primer (P2: 5′-ACGGGCGG TGTGTRC-3′) (Sigma-Aldrich). The amplified product was sequenced and sequence comparison with databases was performed using BLAST through the NCBI server [41]. The isolated bacterium was identified as* Bacillus* sp. IND12. The 16S rDNA sequence was submitted in GenBank database under the accession number KF638633.

### 2.3. Maintenance of Culture

*Bacillus* sp. IND12 was grown on nutrient agar slant which consisted of 0.5% (w/v) peptic digest of animal tissue, 0.15% (w/v) beef extract, 0.15% yeast extract, 0.5% sodium chloride, and 1.5% (w/v) agar. The nutrient agar slant was incubated at 37°C for 24 h and the strain was stored at −20°C as the glycerol stock.

### 2.4. Inoculum Preparation

Seed culture for fibrinolytic enzyme production was prepared by inoculating a loopful culture of this strain into nutrient broth medium (peptic digest of animal tissue, 5.0; beef extract, 1.5; yeast extract, 1.5; and sodium chloride, 5.0; [g/L]). The 250-mL culture flask was further incubated at 37°C for 24 h in a rotary shaker at 175 rpm and used as inoculum.

### 2.5. Screening of Different Agroindustrial Waste Materials for the Production of Fibrinolytic Enzyme by Solid-State Fermentation

The substrates such as wheat bran and rice bran were collected from the local market. Green gram husk, tapioca peel, and banana peel were processed separately, and cow dung was collected from the farm house. All substrates were dried for seven days, powdered, and sieved (1.0–1.5 mm in size). 5.0 g of substrates was taken in Erlenmeyer flask individually and Tris buffer (pH 8.0, 0.1 M) was added to maintain the moisture level (100%, v/w). The contents of the Erlenmeyer flasks were mixed and autoclaved at 15 lbs for 15 min. The Erlenmeyer flasks were inoculated with 10% inoculum (0.810 ± 0.250 OD at 600 nm) and incubated at 37°C for 72 h. 50 mL double-distilled water was added to the fermented medium. It was placed in an orbital shaker for 30 min at 150 rpm at room temperature (30 ± 2°C). The mixed slurry was squeezed through a cheese cloth and centrifuged at 4°C for 20 min at 10,000 rpm. The cell-free clear supernatant was used as the source of enzyme.

### 2.6. Fibrinolytic Enzyme Assay

Fibrinolytic enzyme was assayed by the hydrolysis of fibrin [42]. The reaction mixture contained 2.5 mL of fibrin (1.2%, w/v), 2.5 mL of Tris-HCl (0.1 M) containing 0.01 M CaCl_2_ (pH 7.8), and a suitable amount of enzyme. The incubation was carried out for 30 min (37°C), and the measurement time fell within the linear portion of the progress curve. The reaction was stopped by adding 5 mL trichloroacetic acid (TCA) containing 0.22 M sodium acetate and 0.33 M acetic acid. The absorbance of the supernatant was measured at 275 nm. One fibrinolytic enzyme unit was defined as the amount of sample that gave an increase in absorbency at 275 nm equivalent to 1 *μ*g of tyrosine per min at 37°C.

### 2.7. Protease Assay

Casein was used as the substrate for determining the protease activity. The reaction mixture contained casein (prepared in 0.05 M of Tris-HCl buffer, pH 8.0) and 0.1 mL of enzyme solution. This mixture was incubated for 30 min at 37°C and the measurement time fell within the linear portion of the progress curve. The reaction was stopped by adding 2.5 mL of TCA (0.11 M), and the mixture was centrifuged at 10,000 ×g (10 min). The optical density of the solution was read against sample blank at 280 nm. One unit of the protease activity was defined as 1 *μ*g of tyrosine liberated per minute under assay conditions. The total protein content of the crude enzyme was determined as described by Lowry et al. [43].

### 2.8. Screening of Variables for the Production of Fibrinolytic Enzyme by One-Variable-at-a-Time Approach

The “one-variable-at-a-time” strategy was applied in order to optimize the various nutrient parameters required for fibrinolytic enzyme production. Cow dung (5 g) was used as the substrate for fibrinolytic enzyme production in SSF. Each subsequent nutrient factor was examined after taking into account the previously optimized factor. In the present study, the medium components such as carbon sources (1% [w/w], sucrose, maltose, xylose, glucose, trehalose, and starch), nitrogen sources (1% [w/w], casein, yeast extract, urea, gelatin, peptone, and beef extract), and inorganic salts (0.1% [w/w], calcium chloride, ammonium chloride, ferrous sulfate, ammonium sulfate, disodium hydrogen phosphate, sodium nitrate, magnesium chloride, and sodium dihydrogen phosphate) were tested. The enzyme was extracted as described earlier, and enzyme assay was carried out. All experiments were performed in triplicate and expressed as mean ± standard deviation. Analysis of variance (ANOVA) was carried out to find the significance of variance.

### 2.9. Selection of Significant Variables by 2^5^ Full-Factorial Design

Two-level full-factorial design was employed to elucidate the relative importance of various medium components affecting fibrinolytic enzyme production. To determine the significant nutrient and physical factors for fibrinolytic enzyme production, five variables were selected. The variables used were moisture, pH, sucrose, peptone, and MgSO_4_ at two levels (i.e., −1 and +1). The software Design-Expert (version 9.0.6) was used to design and analyze the data. The mean value of “−1 and +1” was used to evaluate the average response of this model design.

(2) Y=α0+∑αixii+∑αijxixjij+∑αijkxixjxkijk+∑αijklxixjxkxlijkl+∑αijklmxixjxkxlxmijklm,

where *Y* is the response, *α*_*ij*_ = *ij*th, *α*_*ijk*_ = *ijk*th, *α*_*ijkl*_ = *ijkl*th, and *α*_*ijklm*_ = *ijklm*th interaction coefficients, *α*_0_ was an intercept, and *α*_*i*_ was the *i*th linear coefficient.

### 2.10. Optimization of Medium Components by RSM

CCRD was employed to determine the best combination for fibrinolytic enzyme production. A total number of 20 sets of experimental runs with different combination of physical and nutrient factors were performed (14 noncentral points and 6 central points). Each variable was tested at five levels (−*α*, −1, 0, +1, and +*α*). The other factors such as peptone, pH, and inoculum were set at middle level. The fibrinolytic enzyme activity was assayed in triplicate, and the average value was reported as response (*Y*). The results obtained from this model were subjected to analysis of variance (ANOVA) for analyzing the regression coefficient. The results were best fitted with the second-order polynomial equation by a multiple regression technique. The combination of variables that showed the maximum response was determined, and the fitted model was validated. The suitability of the polynomial model equation was judged by determining *R*^2^, and its significance was determined by *F*-test.

The second-order polynomial describing a system with three factors is as follows:

(3) $$\begin{array}{c} Y=\beta_{0}+\sum \overset{3}{\underset{i=1}{\beta_{i}X_{i}}}+\sum \overset{3}{\underset{i=1}{\beta_{ii}{X_{i}}^{2}}}+\sum \overset{3}{\underset{ij=1}{\beta_{ij}X_{ij}}}, \end{array}$$

where *Y* is the response (fibrinolytic activity), *β*_0_ and *β*_*i*_ are the offset and coefficients of linear terms, respectively, and *β*_*ii*_ and *β*_*ij*_ are the coefficients of square terms and coefficients of interactive terms, respectively. *X*_*i*_s were *A*, *B*, and *C*; *X*_*ij*_s were *AB*, *AC*, and *BC* (*A*-coded value of moisture, *B*-coded value of sucrose, and *C*-coded value of MgSO_4_).

Design-Expert 9.0.6 was the statistical software used to design and analyze the experimental data. The fitted quadratic model of the experimental trial was expressed in the form of 3D response surface graphs. These 3D graphs illustrate the main effect and interactive effects of the independent factors on the dependent factor. The predicted conditions were used to perform experiments in order to validate the generated model.

### 2.11. Digestion of Proteins

Crude enzyme was appropriately diluted at 50 U/mL concentrations in 50 mM Tris-HCl buffer (pH 8.0). In the present study, crude enzyme was incubated with goat blood clot (1.0 g), egg white (1.0 g), chicken skin (1.0 g), and bovine serum albumin (1%, w/v) for 24 h at room temperature (30 ± 2°C). The substrate that was incubated with buffer was considered as control. The remaining total protein was estimated and the digested protein content was calculated (%).

## 3. Results and Discussion

Fibrinolytic treatment such as administration of urokinase intravenously is widely used but the enzyme is expensive, and there is risk of internal hemorrhage within the intestinal tract. So, research has been pursued to improve the thrombolytic therapy in terms of efficacy and specificity. In the present study many potent bacterial isolates were isolated from the soil sample, fishes, and cooked rice for the production of fibrinolytic enzymes.

### 3.1. Screening of Bacterial Isolate for Fibrinolytic Enzyme Production

**Figure 1:**
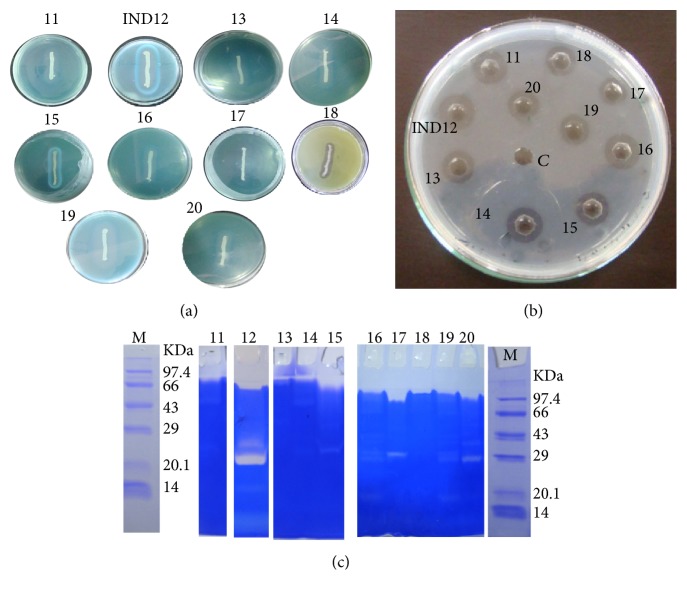
Screening of bacterial isolates for proteolytic activity. The strain IND12 showed a large halo zone compared to other tested bacterial isolates (a). Screening of fibrinolytic enzyme from the selected bacteria. Fibrinolytic activity appeared as a halo zone from the protease positive strains 11–20 (b). Fibrinolytic enzyme activity on SDS-PAGE. The strain IND12 showed potent activity (c).

The fibrinolytic enzyme produced by the bacterial isolate was primarily determined by skimmed milk agar plates. *P*_*z*_ value was found to be much less for* Bacillus* sp. IND12 (0.20) and this low *P*_*z*_ value indicated good protease production (Figure 1(a)). Fibrinolytic enzyme is a subclass of proteases and has an ability to degrade fibrin substrate [44]. Hence, in this study, the protease secreting ability of the bacterial isolates was initially screened using skimmed milk agar plates. All the selected 10 isolates showed fibrinolytic activity on this plate (Figure 1(b)). Fibrin plate method is the most suitable method for the determination of fibrinolytic activity from bacteria, fungi, and other sources. In addition to these, fibrin zymography and in vitro blood clot lytic activity of the crude enzyme of all ten isolates were studied. Most of the selected bacterial isolates synthesized more than one fibrinolytic enzyme. The molecular weight of the fibrinolytic enzyme ranges from 15 to 90 kDa (Figure 1(c)). The* Bacillus* sp. such as* B. subtilis* 168,* B. subtilis* CH3–5,* B*.* amyloliquefaciens *CH51,* B. amyloliquefaciens *CH86, and* B. licheniformis* CH3–17 produced more than one fibrinolytic enzyme at this range [45]. The fibrinolytic enzymes gradually dissolved blood clot within 24 h of incubation at room temperature (30 ± 2°C). Most of the bacterial fibrinolytic enzymes dissolved blood clot completely (Table 1). These results suggested that fibrinolytic enzymes had obvious effect on dissolving blood clot. This kind of clot lysis was reported with* B. subtilis* LD-8547 fibrinolytic enzymes [46]. Based on these four steps' screening procedure, IND12 showed potent activity. The selected organism was rod shaped and oxidase- and catalase-positive. It hydrolyzed casein and starch and tested negative for gelatin hydrolysis. It had tested negative for indole formation, citrate utilization, and H_2_S production. The proteolytic enzyme production by any bacterial isolate mainly depends on the growth media and other physical factors. However, the optimum concentration of the medium varies from strain to strain [47]. Hence, it is important to screen the medium components and physical factors for the new bacterial isolates.

### 3.2. Cow Dung Is a Potential Medium for the Production of Fibrinolytic Enzyme

The bacterial isolate,* Bacillus* sp. IND12, utilized all tested agroindustrial residues for the production of fibrinolytic enzyme. This organism utilized cow dung effectively for its growth and fibrinolytic enzyme production. Enzyme production was high in the cow dung substrate (687 ± 6.5 U/g) (Table 2). The selection of an ideal substrate for enzyme production in SSF depends on several factors including availability and cost [9] and nutritive value of the medium [29]. Cow dung is one of the unexploited and most abundant feedstock for fibrinolytic enzyme production. It contains cellulose (35.4–37.6%), hemicelluloses (32.6–34.1%), ash (13.3–13.4%), nitrogen (1.2–1.4%), and other essential nutrients such as Mg, Ca, Zn, S, Cu, B, and Mn [48, 49]. The carbon, nitrogen, and other nutrients' content in cow dung are an indication that it could be a good nutrient source for the microbes [50]. The agroindustrial residues such as rice chaff [51], shrimp shells [4], cane molasses [52], and wheat bran [7] were used as the substrate for the production of fibrinolytic enzyme. The availability of cow dung substrate is higher than most of the reported substrates. Considering the availability and cost, cow dung is an ideal medium for the production of fibrinolytic enzyme from* Bacillus* sp. IND12. The growth of this strain on cow dung is important for the production of various industrially useful enzymes in cheap cost.

### 3.3. Effect of Carbon, Nitrogen, and Ionic Sources

**Figure 2:**
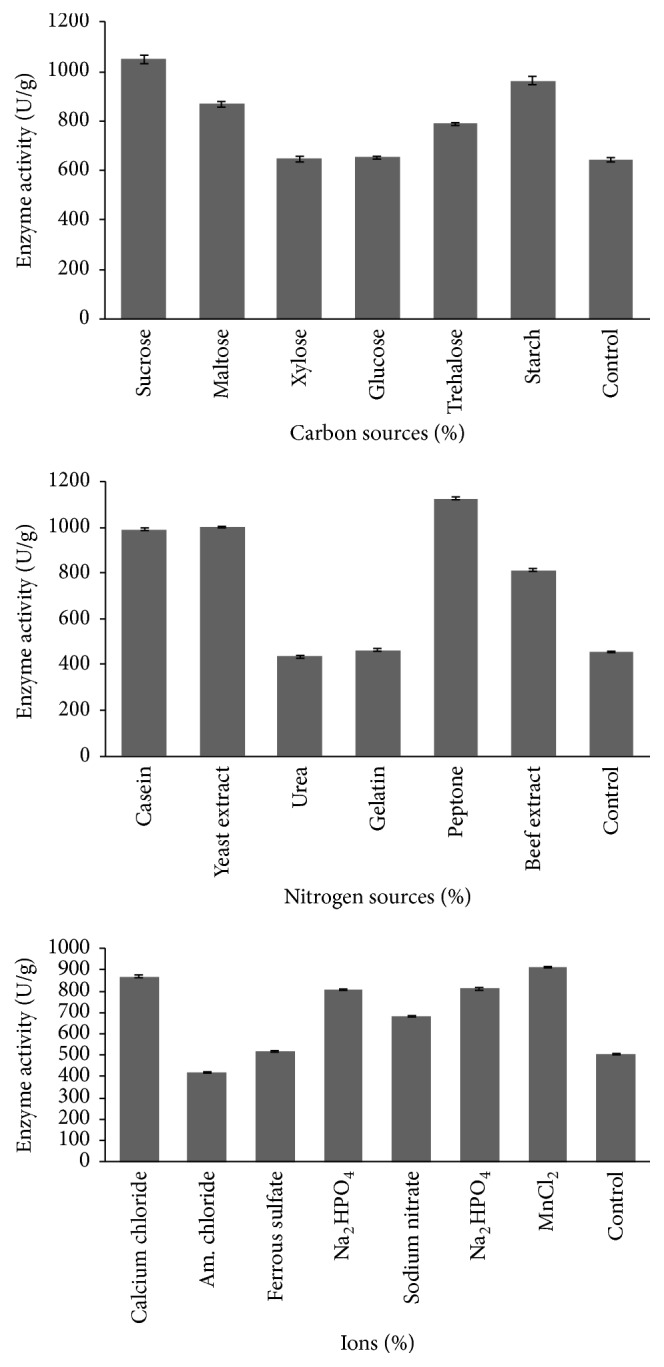
Effect of carbon sources, nitrogen sources, and ions on fibrinolytic enzyme production by* Bacillus* sp. IND12. These nutrient sources were supplemented individually with cow dung substrate, inoculated with 10% inoculum (0.810 ± 0.250 OD at 600 nm), and incubated at 37°C for 72 h.

To screen different carbon sources, 1% carbon source (maltose, glucose, sucrose, xylose, starch, and trehalose) was supplemented with cow dung substrate. This medium was inoculated with 10% inoculum (0.810 ± 0.250 OD at 600 nm) and incubated for 72 h. All tested carbon sources enhanced fibrinolytic enzyme production (Figure 2). The influence of different carbon sources on fibrinolytic enzyme production was found to be statistically significant (*P* < 0.00001) by the one-way ANOVA. The lowest fibrinolytic enzyme activity was obtained with xylose (648 ± 11.5 U/g) and highest enzyme activity was observed with sucrose (1049 ± 17.43 U/g). Similar results were obtained in the study of Liu et al. [25] in the screening of the carbon sources on the fibrinolytic activity of* Bacillus natto* NLSSE. Considering the cheap cost and enzyme production, sucrose was chosen as the carbon source for further optimization study. To screen different nitrogen sources, 1% casein, peptone, beef extract, yeast extract, urea, and gelatin were supplemented with cow dung substrate. The lowest fibrinolytic activity was registered with urea (435 ± 3.87 U/g) and the highest with peptone (1127 ± 1.38 U/g) (Figure 2). The influence of different nitrogen sources on fibrinolytic enzyme production was found to be statistically significant (*P* < 0.00001) by the one-way ANOVA. Similar results were obtained with* Bacillus subtilis* RJAS19 [53] and* Streptomyces* sp. [54]. From this result, peptone was chosen as the nitrogen source for further optimization study. The culture medium was supplemented with 0.1% ions such as calcium chloride, ammonium chloride, ferrous sulfate, ammonium sulfate, disodium hydrogen phosphate, sodium nitrate, and sodium dihydrogen phosphate. Enzyme production was found to be high in the medium containing MgSO_4_ as the ionic source. The ions such as Ca^2+^ and Mn^2+^ also enhanced the production of fibrinolytic enzyme, and enzyme activity was 867.4 ± 4.9 and 912.6 ± 3.8 U/g, respectively (Figure 2). The influence of different ionic sources on fibrinolytic enzyme production was found to be statistically significant (*P* < 0.00001) by the one-way ANOVA. In this study, Ca^2+^ ions also positively regulated enzyme production. The positive effect of divalent ions such as Mg^2+^ and Mn^2+^ was reported by Pillai et al. [55] with* Bacillus subtilis* P13. The stimulating effect of CaCl_2_ was also stated by Mabrouk et al. [56]. The presence of other components at 0.1% level did not positively influence the enzyme production.

### 3.4. Studies on the Effect of Process Variables on Fibrinolytic Enzyme Production by 2^5^ Full-Factorial Design

The significance of five medium components, namely, moisture (*A*), pH (*B*), sucrose (*C*), peptone (*D*), and MgSO_4_ (*E*), for the production of fibrinolytic enzyme was elucidated as given by 2^5^ full-factorial design. The factors and their levels were described in detail (Table 3). The 2^5^ full-factorial experiment showed wide variation of enzyme activity. In the present study, all factors positively regulated fibrinolytic enzyme production. The fibrinolytic enzyme production by* Bacillus* sp. IND12 varied from 292 to 3949 U/g in 32 experiments, which suggests the significance of medium components and their concentration on fibrinolytic enzyme production. The results showed that run number 3 resulted in the highest fibrinolytic enzyme production (3949 U/g), followed by medium composition in run 14 (3881 U/g) (Table 4). The observed mean response of this two-level full-factorial design was 1621.13 U/g. The main effect, *F* value, and *P* value of each factor are given in Table 5. According to these ANOVA results, the five variables such as *A*, *B*, *C*, *D*, and *E* were statistically significant on fibrinolytic enzyme production. Among the variables, the most significant variable was moisture, which had highest correlation coefficient compared to the other tested factors. The linear, interactive, and quadratic coefficients such as *AC*, *AE*, *BC*, *BE*, *CD*, *ABC*, *ABD*, *ABE*, *ACE*, *BCE*, *BDE*, *CDE*, *ABCD*, *ABCE*, *BCDE*, and *ABCDE* were also statistically significant. The regression equation involving the coded factors is

(4) Enzyme activityY=+1654.88+180A+88.25B+214.19C+141.31D+265.88E+215.69AC−98.75AE−249.06BC−166.06BD+115.25BE+234.25CD+149.56ABC+116.56ABD−123.5ABE+79.56ACE−101.94BCE−412.06BDE+79.37CDE−106ABCD+164.44ABCE−228.13BCDE−233.62ABCDE.

Among the factors, the coefficient estimate was high for moisture, sucrose, and MgSO_4_. Hence these three factors were selected for further studies. The middle value of the other two factors (0.5% (w/w) peptone and pH 8.0) was used for CCRD.

### 3.5. Response Surface Methodology

**Figure 3:**
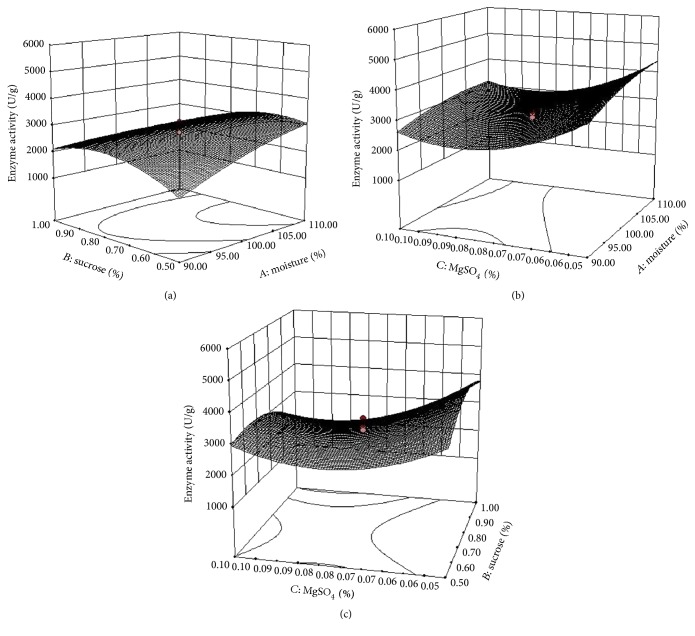
Response surface curve showing the effect of moisture and sucrose (a), moisture and MgSO_4_ (b), and sucrose and MgSO_4_ (c) on the fibrinolytic activity of* Bacillus* sp. IND12. The other factors (pH and peptone) were kept at middle level.

A CCRD was employed to find the interactions among significant independent factors (moisture, sucrose, and MgSO_4_). Each variable was analyzed at five coded levels (−*α*, −1, 0, +1, +*α*) (Table 6). Enzyme assay was carried out and the experimental result was taken as response *Y* (Table 7). The *F*-test for an ANOVA was generated to elucidate the significance of the model and understand the reliability of the regression model. The selected variables, moisture, sucrose, and MgSO_4_, were found to be significant (*P* < 0.05) (Table 8). The interactive effects such as *AB*, *BC*, *A*^2^, *B*^2^, and *C*^2^ were also statistically significant. The determinant coefficient termed *R*^2^ was calculated to be 0.9933 for fibrinolytic enzyme production by* Bacillus* sp. IND12, suggesting 99.33% of the variability in the response. The final equation in terms of coded factor can be written as follows:

(5) Fibrinolytic enzymeY=+2921.44+340.22A−147.18B−543.64C−439.62AB−75.87AC−340.62BC−175.81A2−588.93B2+707.19C2,

where *Y* was the response of fibrinolytic yield and *A*, *B*, and *C* were the coded terms for independent variables of moisture, sucrose, and MgSO_4_, respectively. The interactions of variables in the model were demonstrated as 3D response surface plots (Figures 3(a)–3(c)). The *R*^2^ value of the predicted model was 0.9848, which was reasonable in agreement with adjusted *R*^2^ of 0.9874. The adequate precision reflects on the signal-to-noise ratio; the ratio of 55.662 indicates adequate signal of the model. The response surface curve shows the interaction between moisture and sucrose. Enzyme production was maximum at higher moisture content and decreased at lower moisture content (Figure 3(a)). The moisture content of SSF medium takes different optimum values with differing substrates. It was reported that 40% was optimum for the production of proteolytic enzyme using wheat bran and lentil husk as the substrate [57]; however, 140% was registered as the optimum moisture content for green gram husk substrate for protease production [47]. In earlier reports of fibrinolytic enzyme production, many organic carbon sources have been used in the medium, including sucrose for* Paenibacillus* sp. IND8 [58], whereas glucose and soybean flour were used with* Streptomyces* sp. [59]. The data suggest that cow dung is a promising substrate for use in fibrinolytic enzyme production.

The three-dimensional response surface for the interaction of MgSO_4_ and moisture is represented (Figure 3(b)). The fibrinolytic enzyme yield was found to be increased with higher concentration of moisture. The results showed that fibrinolytic enzyme production was most affected by moisture levels compared to nutrient factors. In SSF, moisture content of the medium is critically important. The interaction of sucrose and MgSO_4_ indicated that fibrinolytic enzyme yield was significantly affected by interaction between these two factors (Figure 3(c)). The optimized medium (109.73% moisture, 0.57% sucrose, and 0.093% MgSO_4_) showed 4143 ± 12.31 U/g material, which was more than fourfold the unoptimized medium (978 ± 36.4 U/g). Deepak et al. [60] reported similar optimization with RSM with* Bacillus subtilis*, which resulted in a twofold increase in fibrinolytic enzyme production. RSM was also employed for* Bacillus* sp. strain AS-S20-I [61] and* Paenibacillus* sp. IND8 [58], and 4-fold and 4.5-fold enzyme production was reported.

### 3.6. Validation of the Predicted Model

Validation experiments to test the appropriateness of the model were conducted with the strain IND12 under the following conditions: 109.73% moisture, 0.57% sucrose, and 0.093% MgSO_4_. Fibrinolytic enzyme production reached the maximum of 4143 ± 12.31 U/g after 72 h incubation, which was highly comparable with the predicted value (4168.68 ± 36.4 U/g). The observed and predicted values went hand in hand, indicating the model validation.

### 3.7. Digestion of Proteins from Natural Sources by* Bacillus* sp. IND12 Protease

The ability of crude protease to digest some natural proteins was tested. The enzyme digested goat blood clot completely (100 ± 0.92% solubility) within 24 h of incubation at room temperature (30°C ± 2°C). These results showed that the fibrinolytic enzyme of* Bacillus* sp. IND12 can convert the insoluble forms of goat blood clot into soluble form. It also hydrolyzed 29 ± 4.9% bovine serum albumin within 12 h of incubation. This enzyme digested coagulated egg white (100 ± 0.62% solubility) and also hydrolyzed chicken skin (83 ± 3.6% solubility). The study suggests the usefulness of this enzyme for various applications including collagen replacement therapy and waste treatment. These kinds of proteolytic activity were registered previously with* Pseudomonas aeruginosa* PD100 [62] and* Bacillus* RV.B2.90 [63]. The activity of the protease on egg protein, chicken skin, and blood clot indicates its importance in industrial application, waste treatment, and medicine.

## 4. Conclusion

To conclude, four-step screening was successful to evaluate the potent fibrinolytic enzyme-producing bacterial isolate. Cow dung was used as the cheap substrate for the production of fibrinolytic enzyme from* Bacillus* sp. IND12. The optimum process parameters were as follows: 109.73% moisture, 0.57% sucrose, and 0.093% MgSO_4_. At these conditions, fibrinolytic enzyme production reached the maximum of 4143 ± 12.31 U/g after 72 h incubation, which was highly comparable with the predicted value (4168.68 ± 36.4 U/g). This enzyme hydrolyzed goat blood clot, chicken skin, egg white, and bovine serum albumin, which suggested its applications in clinical and waste water treatment. The bioprocessing of this low cost substrate can minimize the production cost of enzyme. The enhanced yield of fibrinolytic enzyme by* Bacillus *sp. IND12 using cow dung substrate could be useful for the production of enzyme in an industrial scale.

## Figures and Tables

**Table 1:** In vitro blood clot lytic activity of fibrinolytic enzymes from the bacterial isolates.

Bacterial strains	% of blood clot lysis
11	100
12 (IND12)	100
13	87 ± 12
14	79 ± 7.1
15	100
16	98.3 ± 14.8
17	63.7 ± 10.9
18	53.4 ± 1.2
19	100
20	100

**Table 2:** Effect of agroresidues on fibrinolytic enzyme production in SSF.

Agroresidues	Fibrinolytic activity (U/g)
Banana peel	85
Cow dung	687
Green gram husk	493
Rice bran	273
Tapioca peel	384
Wheat bran	640

**Table 3:** Variables and their levels for 2^5^ full-factorial design.

Symbol	Variable name	Units	Coded levels	
			−1	+1
*A*	Moisture	%	90	120
*B*	pH		7	9
*C*	Sucrose	%	0.1	1
*D*	Peptone	%	0.1	1
*E*	MgSO_4_	%	0.01	0.1

**Table 4:** Two-level full-factorial design for selection of significant process variables for fibrinolytic enzyme production from *Bacillus *sp. IND12.

Run	Moisture (*A*)	pH (*B*)	Sucrose (*C*)	Peptone (*D*)	MgSO_4_ (*E*)	Enzyme activity (U/g)
1	1	1	1	1	1	1920
2	−1	1	1	1	1	1419
3	1	−1	1	1	1	3949
4	−1	−1	1	1	1	3122
5	1	1	−1	1	1	1473
6	−1	1	−1	1	1	1949
7	1	−1	−1	1	1	1166
8	−1	−1	−1	1	1	1521
9	1	1	1	−1	1	3184
10	−1	1	1	−1	1	1466
11	1	−1	1	−1	1	1032
12	−1	−1	1	−1	1	1066
13	1	1	−1	−1	1	1702
14	−1	1	−1	−1	1	3881
15	1	−1	−1	−1	1	1590
16	−1	−1	−1	−1	1	292
17	1	1	1	1	−1	3038
18	−1	1	1	1	−1	1377
19	1	−1	1	1	−1	1642
20	−1	−1	1	1	−1	1490
21	1	1	−1	1	−1	1638
22	−1	1	−1	1	−1	933
23	1	−1	−1	1	−1	1100
24	−1	−1	−1	1	−1	1002
25	1	1	1	−1	−1	823
26	−1	1	1	−1	−1	439
27	1	−1	1	−1	−1	2530
28	−1	−1	1	−1	−1	1408
29	1	1	−1	−1	−1	1509
30	−1	1	−1	−1	−1	1139
31	1	−1	−1	−1	−1	1062
32	−1	−1	−1	−1	−1	1094

**Table 5:** Results of ANOVA for the two-level full-factorial design.

Source	Sum of squares	df	Mean square	*F* value	*P* value
Model	2.50*E* + 07	22	1.13*E* + 06	85.09	<0.0001
*A*-moisture	1.04*E* + 06	1	1.04*E* + 06	77.79	<0.0001
*B*-pH	2.49*E* + 05	1	2.49*E* + 05	18.7	0.0019
*C*-sucrose	1.47*E* + 06	1	1.47*E* + 06	110.15	<0.0001
*D*-peptone	6.39*E* + 05	1	6.39*E* + 05	47.95	<0.0001
*E*-MgSO_4_	2.26*E* + 06	1	2.26*E* + 06	169.73	<0.0001
*AC*	1.49*E* + 06	1	1.49*E* + 06	111.7	<0.0001
*AE*	3.12*E* + 05	1	3.12*E* + 05	23.41	0.0009
*BC*	1.99*E* + 06	1	1.99*E* + 06	148.94	<0.0001
*BD*	8.83*E* + 05	1	8.83*E* + 05	66.21	<0.0001
*BE*	4.25*E* + 05	1	4.25*E* + 05	31.89	0.0003
*CD*	1.76*E* + 06	1	1.76*E* + 06	131.75	<0.0001
*ABC*	7.16*E* + 05	1	7.16*E* + 05	53.71	<0.0001
*ABD*	4.35*E* + 05	1	4.35*E* + 05	32.62	0.0003
*ABE*	4.88*E* + 05	1	4.88*E* + 05	36.62	0.0002
*ACE*	2.03*E* + 05	1	2.03*E* + 05	15.2	0.0036
*BCE*	3.33*E* + 05	1	3.33*E* + 05	24.95	0.00017
*BDE*	5.43*E* + 06	1	5.43*E* + 06	407.68	<0.0001
*CDE*	2.02*E* + 05	1	2.02*E* + 05	15.13	0.0037
*ABCD*	3.60*E* + 05	1	3.60*E* + 05	26.98	0.0006
*ABCE*	8.65*E* + 05	1	8.65*E* + 05	64.92	<0.0001
*BCDE*	1.665*E* + 0.006	1	1.665*E* + 0.006	124.95	<0.0001
*ABCDE*	1.75*E* + 06	1	1.75*E* + 06	131.05	<0.0001
Residual	1.20*E* + 05	9	13327.68		
Cor total	2.51*E* + 07	31			

**Table 6:** Variables and their levels for central composite rotational design.

Variables	Symbol	Coded values				
		−*α*	−1	0	+1	+*α*
Moisture (%)	*A*	83.18	90	100	110	116.82
Sucrose (%)	*B*	0.33	0.5	0.75	1.0	1.17
MgSO_4_ (%)	*C*	0.03	0.05	0.08	0.1	0.12

**Table 7:** The matrix of the CCRD experiment and fibrinolytic enzyme activity.

Run	Moisture (*A*)	Sucrose (*B*)	MgSO_4_ (*C*)	Enzyme activity (U/g)
1	−1	1	1	1198
2	0	0	0	3047
3	0	0	0	2740
4	1	1	−1	3508
5	0	0	0	2890
6	0	−1.682	0	1509
7	1	−1	1	3505
8	0	0	1.682	3972
9	0	0	0	2754
10	−1	−1	1	2142
11	0	0	−1.682	5916
12	1	−1	−1	4048
13	1.682	0	0	3032
14	−1.682	0	0	1861
15	0	1.682	0	1047
16	1	1	1	1670
17	−1	−1	−1	2314
18	0	0	0	2940
19	−1	1	−1	3600
20	0	0	0	3150

**Table 8:** Results of ANOVA for the CCRD.

Source	Sum of squares	df	Mean square	*F* value	*P* value
Model	2.247*E* + 007	9	2.497*E* + 006	165.97	<0.0001
*A*-moisture	1.581*E* + 006	1	1.581*E* + 006	105.07	<0.0001
*B*-sucrose	2.958*E* + 005	1	2.958*E* + 005	19.66	0.0013
*C*-MgSO_4_	4.036*E* + 006	1	4.036*E* + 006	268.26	<0.0001
*AB*	1.546*E* + 006	1	1.546*E* + 006	102.76	<0.0001
*AC*	46056.13	1	46056.13	3.06	0.1108
*BC*	9.282*E* + 005	1	9.282*E* + 005	61.69	<0.0001
*A* ^2^	4.454*E* + 005	1	4.454*E* + 005	29.60	0.0003
*B* ^2^	4.998*E* + 006	1	4.998*E* + 006	332.21	<0.0001
*C* ^2^	7.207*E* + 006	1	7.207*E* + 006	479.03	<0.0001
Residual	1.505*E* + 005	10			
Lack of fit	20173.51	5	4034.702	2.98	0.131
Pure error	1.303*E* + 005	5			
Cor total	2.263*E* + 007	19			
